# Do reasons for undergoing bariatric surgery influence weight loss and health-related quality of life?–A Swedish mixed method study

**DOI:** 10.1371/journal.pone.0275868

**Published:** 2022-10-10

**Authors:** Maria Jaensson, Emma Josefsson, Erik Stenberg, Karuna Dahlberg

**Affiliations:** 1 Faculty of Medicine and Health, School of Health Sciences, Örebro University, Örebro, Sweden; 2 School of Medical Sciences, Örebro University, Örebro, Sweden; 3 Department of Surgery, Faculty of Medicine and Health, Örebro University, Örebro, Sweden; Public Library of Science, UNITED KINGDOM

## Abstract

**Background:**

A wish for improved health or avoidance of ill health is often given as reason for wanting to undergo bariatric surgery. How such reasons relate to postoperative outcome is unclear.

**Objective:**

The aim was to explore Swedish patients’ reasons for undergoing bariatric surgery. Also, we wanted to analyze if there were sex and age differences and associations with weight loss and health-related quality of life (HRQoL).

**Settings:**

This was a single-center study conducted at a university hospital.

**Method:**

Data on 688 patients (528 women and 160 men) including a free text response was analyzed inductively and deductively using predefined statements and was merged with data from the Scandinavian Obesity Surgery Registry. All data was analyzed using descriptive and analytic statistics.

**Result:**

The most common reason for undergoing bariatric surgery was pain in different body parts. A wish for an improved medical condition was reported by most patients (59%, n = 408), followed by physical limitations making daily life difficult (42%, n = 288). Men and women reported similar reasons. Younger patients were more distressed about physical appearance (p = 0.001) and older patients wanted to improve their medical condition (p = 0.013). Health-related quality of life improved irrespective of reasons for undergoing surgery.

**Conclusion:**

The most reported reasons for undergoing bariatric surgery were a wish for improved medical condition and to make daily life easier. Factors associated with the decision for surgery showed that there were few sex differences, but age seemed to be a factor. The HRQoL trajectory showed improvement regardless of reasons for undergoing surgery.

## Introduction

Bariatric surgery decreases morbidity and mortality [1], and is an effective treatment for sustainable weight loss [2]. The decision to undergo bariatric surgery is often a long process taking several years and for some patients is their last option [3, 4]. Reasons for undergoing surgery vary; worsened health and low energy levels that impact physical activity [5], as well as avoidance of sequelae/comorbidities [6], have been reported in previous research.

Obesity is associated with low preoperative health-related quality of life (HRQoL) [7]. Long- term follow-up has shown that physical HRQoL is improved after surgery and the improvement lasts even longer than 12 years [8]. Mental HRQoL has a different trajectory, and an improvement is seen in the first years following surgery but is not maintained in the long term [8]. In a Swedish bariatric surgery population, HRQoL was lower in women and younger patients 2 years after surgery. Patients aged <30 years experienced the least improvement in HRQoL, and women experienced a smaller improvement in quality of life (QoL) compared to men. This lower improvement in HRQoL after surgery could be due to the fact that these individuals have different expectations of surgical outcome [9].

The aim of this study was to explore Swedish patients’ reasons for undergoing bariatric surgery. Also, we wanted to analyze if there were sex and age differences in the decision to undergo surgery, and associations with weight loss and HRQoL.

## Method

This study was a retrospective cohort-based study with a convergent design [10]. Ethical approval was sought from and granted by the Swedish Ethical Review Authority (ref: 2020–00936) with the requirement for informed consent being waived. In accordance with Swedish legislation, all participants were informed about quality registry and that the data will be used in clinical research, giving the patients the right to deny participation at any time (“opt-out”).

### Data collection

All patients operated with primary gastric bypass procedure or a sleeve gastrectomy (SG) operation at a University Hospital from 2013 to 2017 were considered for inclusion in this study. As part of the preoperative evaluation, patients were asked to respond to a questionnaire including an open-ended question asking them to write, in their own words, why they had sought to undergo bariatric surgery. All additional variables (i.e., sex, age, weight, body mass index (BMI), excess body mass index loss (EBMIL), type of surgery, education level, HRQoL preoperatively and 1 and 2 years after surgery, and weight loss 1 and 2 years after surgery) were based on data from the Scandinavian Obesity Surgery Registry (SOReg).

Health-related QoL was assessed with the RAND Short Form 36 (SF-36/RAND36, hereafter referred to, simply, as “SF-36”), which consists of 36 items grouped into eight scales (bodily pain, emotional role, general health, mental health, physical function, role function, social functioning, and vitality). Scores range from 0 to 100 and a higher score indicates better health status. Two summary scores, the physical component summary (PCS) and the mental component summary (MCS) scores, were analyzed, reflecting overall physical and mental health status [11–13].

### Analysis of qualitative data

The collected responses were mostly written as single words, e.g., *tired* or *heavy*, or short expressions or terms, e.g., *blood pressure*. There were also whole sentences, e.g., “*I’m afraid of dying*,” “*I want to be able to have a child*,” “*I want to find a partner*.” The open-ended question was analyzed inductively by one researcher (MJ) who coded the responses. This was followed by several discussions with one of the coauthors (KD) until consensus was reached. The codes were then further analyzed using a deductive approach and based on the statements originally developed by Libeton et al [14].

The statements [14] are:

- Appearance: I am distressed by my physical appearance, and I feel the need to improve it.
- Medical condition: I want to improve medical conditions associated with my obesity.
- Physical fitness: I lack physical fitness and want to be more active to enjoy life more.
- Health concerns: I am concerned that my health will deteriorate, and my life will be shortened.
- Embarrassment: I am embarrassed socially about my weight.
- Physical limitation: I feel that my physical limitation of obesity makes day to day living very difficult.
- Employment prospects: I want to improve my work ability and/or improve my employment prospects.

Rank order for responses was analyzed using the above statements by Libeton et al [14]. The assumption for this study was that respondents wrote the most important reason first in their free text responses.

### Analysis of quantitative data

Descriptive statistics are presented as mean (± standard deviation (SD)), number, and percent for demographics, characteristics variables, and HRQoL. To compare differences between groups, age was dichotomized based on mean value, with a cutoff of 40 (young vs. old). To investigate differences, chi-squared test, Fisher’s exact test, Mann-Whitney *U*-test, or dependent *t-*test was used depending on data level and whether data was normally distributed or not. A p-value of <0.05 was considered significant. For the calculations, we used IBM SPSS Statistics, v 26 (IBM, Armonk, NY, US).

## Results

Of the 882 patients included in this study, 184 did not respond to the question asking for their reasons for undergoing surgery, and nine were ill. In total, free text responses from 688 patients, 528 women and 160 men, were received and analyzed (Table 1).

**Table 1:** Participant characteristics and demographics.

	Missing	
	n	
**Sex**, n (%)	0	
Women		528 (77)
Men		160 (23)
**Age**, m(SD)	0	40(11.85)
**Weight** m(SD)	0	120(21.03)
**Body mass index**, m (SD)		
Before surgery		42.0 (5.22)
1 year after surgery	27	28.1 (4.50)
2 years after surgery	41	28.4 (4.72)
**Education level**, n (%)	48	
Primary (<9 yrs)		53 (8)
Secondary (10–12 yrs)		460 (67)
Higher (>13 yrs)		127 (19)
**HRQoL**, m (SD)		
Before surgery PCS/MCS	112	36.0 (11.77)/45.6 (13.90)
1 year after surgery PCS/MCS	178/179	51.1 (9.84)/48.2 (11.59)
2 years after surgery PCS/MCS	260	50.7 (9.86)/45.4 (13.31)
**Somatic comorbidity**, n (%)	0	
Depression		68(10)
Diabetes		81 (12)
Dyslipidemia		49 (7)
Hypertension		162 (24)
Sleep apnea		73 (11)
**Type of surgery**, n (%)	0	
Gastric bypass procedure		632 (92)
Sleeve gastrectomy (SG) only		56 (8)

HRQoL = health-related quality of life; MCS = mental component summary; PCS = physical component summary; SD = standard deviation.

### Reasons for undergoing surgery

The most common single word for the whole sample was *pain* in different body parts (n = 336/688), followed by *lack of mobility* (n = 267/688) and then *tiredness* (n = 185/688). There were no significant differences in wording of answers between men and women.

The rank order among Libeton et al´s statements [14] was calculated, with medical condition mentioned first (n = 210/688, 31%), followed by physical limitation (n = 158/688, 23%), health concerns (n = 127/688, 19%), physical fitness (n = 106/688, 15%), appearance (n = 58/688, 8%), employment (n = 16/688, 2%), and embarrassment (n = 13/688, 2%).

A wish to improve their medical condition was the most common response in this study (n = 408/688, 59%) followed by physical limitations making day to day life difficult (n = 288/688, 41%). Significantly more women than men reported a wish to improve their physical fitness (p = 0.01) (Table 2).

**Table 2:** Reasons for undergoing surgery for the whole sample and for men and women separately.

	All, n = 688	Men, n = 160	Women, n = 528	Examples of responses
	n (%)	n (%)	n (%)	
	yes/no	yes/no	yes/no	
Appearance: I am distressed by my physical appearance, and I feel the need to improve it.	182 (27)/506 (73)	33 (21)/127(79)	149 (28)/379(72)	“Hard to look at oneself in the mirror”
				“Low self-esteem”
				“Do not like myself or my body”
Medical condition: I want to improve medical conditions associated with my obesity.	408 (59)/280 (41)	86 (54)/74(46)	322 (61)/206(39)	“Problems with pain, wanting to reduce pain in my back and knees”
				“High blood pressure”
				“Diabetes”
				“Difficulty breathing”
				“I want to be a parent”
Physical fitness: I lack physical fitness and want to be more active to enjoy life more.	280 (41)/408 (59)	51 (32)/109(68)	229(43)/299/57)*	“Problems with mobility and activity and exercise”
Health concerns: I am concerned that my health will deteriorate, and my life will be shortened.	266 (39)/422 (61)	56 (35)/104(65)	210 (40)/318(60)	“I want to avoid comorbidity”
				“I am afraid of serious illness and death”
Embarrassment: I am embarrassed socially about my weight.	86 (12)/602 (88)	14 (9)/147(91)	72 (14)/456/86)	“I avoid all social contact”
				“Social isolation”
				“I don’t dare to meet new people”
Physical limitation: I feel that my physical limitation of obesity makes day to day living very difficult.	288 (42)/400 (58)	60 (38)/100(63)	228 (43)/300(57)	“Everything in my day to day life is difficult”
				“Tiredness”
				“Difficult to sit and sleep comfortably”
Employment prospects: I want to improve my work ability and/or improve my employment prospects.	65 (9)/623 (91)	21 (13)/139(87)	44 (8)/484(92)	“Limited at work”
				“Can’t find a job”
				“I want to be able to work”

*p = 0.01 analyzed with Chi-squared test.

Analyzing differences between different age groups (young vs. old) showed that younger patients were more distressed about their physical appearance (p = 0.001) and older patients wanted to improve their medical condition (p = 0.013). Distress about physical appearance differed significantly between younger and older women (18–39 years compared to 40–73 years, n = 87 (58%) vs. n = 62 (42%), p = 0.007). No other differences were found between men and women in different age groups.

### Weight loss and reasons for surgery

Clinical follow-up with weight measurement was recorded for 662 patients (96.2%) at 1 year, and for 648 patients (94.2%) at 2 years after surgery. Mean BMI loss at 1 year was 13.9 ± 3.97 kg/m^2^, with the corresponding excess BMI loss being 85.4 ± 23.5, and total weight loss 33.0 ± 7.8% of body weight. Mean BMI loss at 2 years after surgery was 13.7 ± 4.44 kg/m^2^, with the corresponding excess BMI loss at 83.1 ± 24.28% and total weight loss at 32.3 ± 8.8% of body weight. No difference in weight loss was seen when data was stratified by reason to undergo surgery (S1 Table).

### Health-related quality of life and reasons for surgery

For the whole group, the PCS score increased significantly from the preoperative value to 1 year (95% CI, -16.08 ˗ -11.61, p = 0.001) and 2 years (95% CI -15.92 ˗ -10.13, p = 0.001) after surgery. The MCS score increased significantly from preoperatively to 1 year (95% CI -5.63˗ -.36, p = 0.02) but not 2 years after surgery.

**Fig 1:**
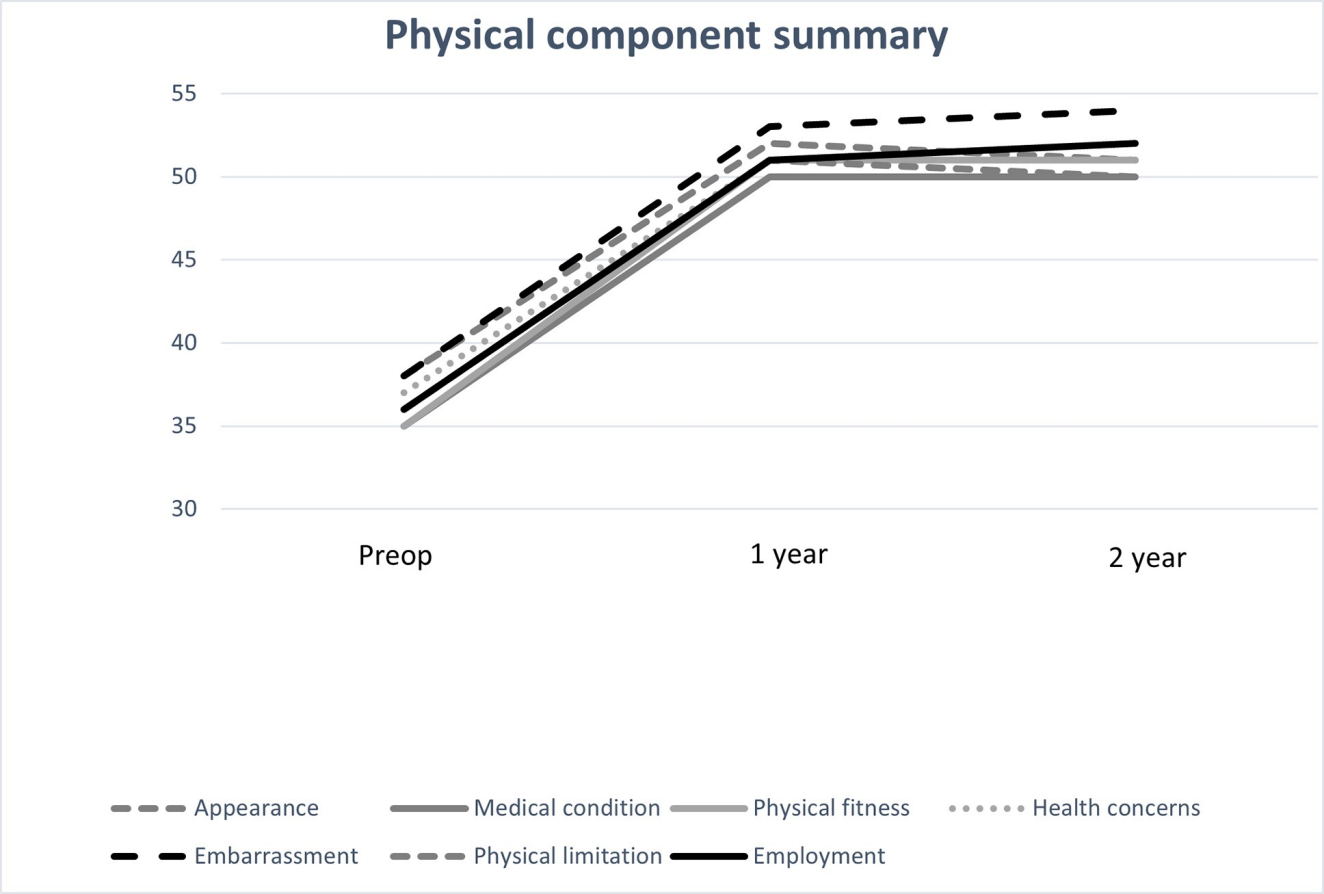
Physical component summary (PCS) scores and reasons for undergoing surgery, analyzed using dependent *t-*test. All categories increased significantly 1-year after surgery (p = 0.001) and 2-years (p = 0.001). Missing: preoperatively n = 112, 1-year n = 178, 2-year n = 260.

**Fig 2:**
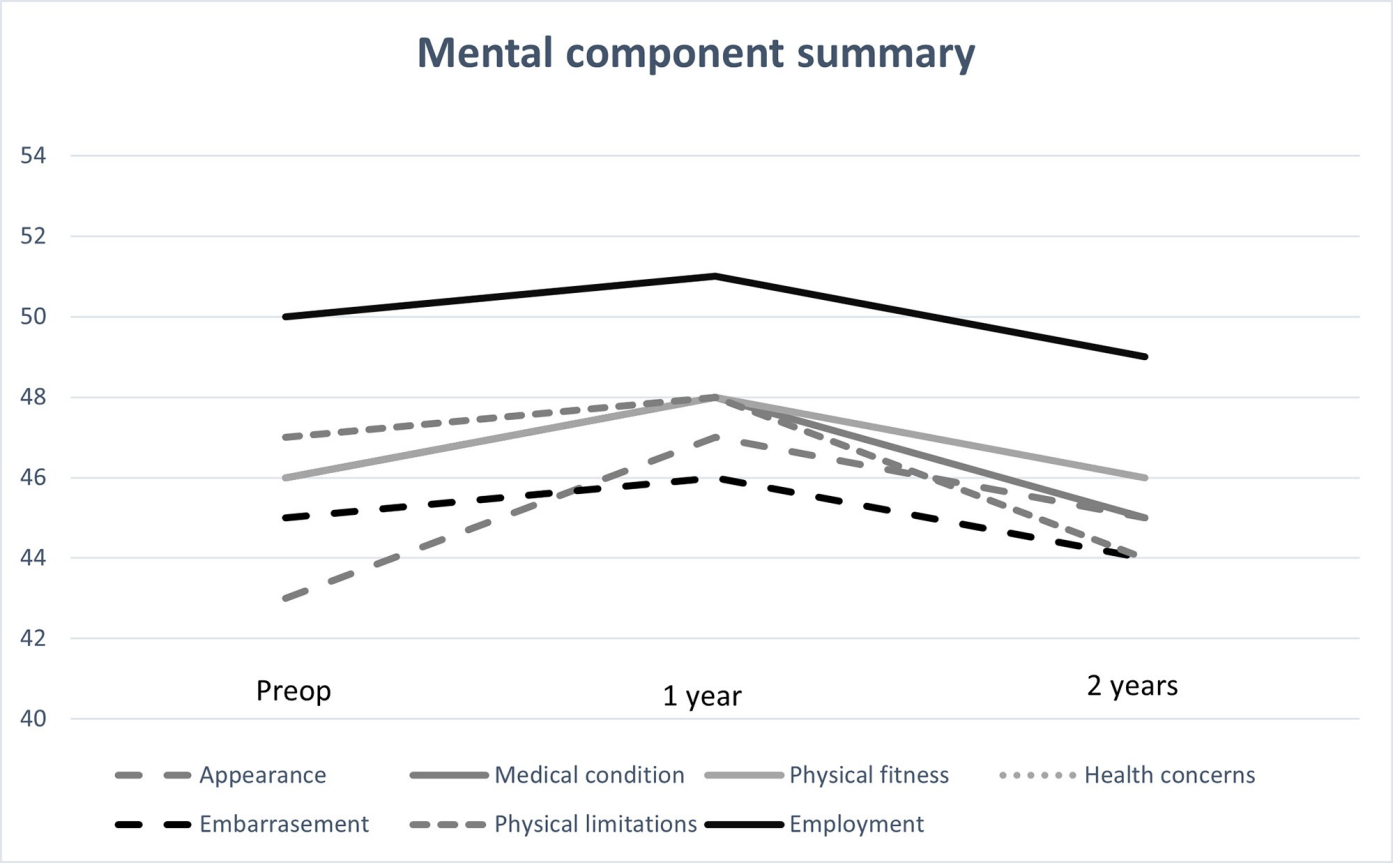
Mental component summary (MCS) scores and reasons for undergoing surgery, analyzed using dependent *t-*test. Significantly increased values 1-year after surgery in categories appearance (p = 0.026) and health concerns (p = 0.01). Missing: preoperatively n = 112, 1-year n = 179, 2-year n = 260.

Regardless of reason for surgery, the PCS score increased significantly from the preoperative value in all categories both at 1 year (p = 0.001) and at 2 years (p = 0.001) after surgery (Fig 1). The MCS score increased in all categories, but the increase was only significant for the preoperative to the 1-year value in the categories appearance (p = 0.026) and health concerns (p = 0.01) (Fig 2).

## Discussion

The expectations of and motivation for patients wanting to undergo bariatric surgery are not fully understood or thoroughly investigated. In this sample of 688 patients, the main reason to undergo bariatric surgery was a wish by patients to improve their medical condition, followed by improved mobility with fewer physical limitations in everyday life. Forty-nine percent of patients reported pain in different body parts. No significant association between reasons for wanting to undergo surgery, sex, and weight loss after surgery was seen. Reasons for wanting to undergo surgery do not appear to serve as a predictor for weight loss. Instead, the ability for short- and long-term weight loss is related to cognitive function, personality, psychiatric well-being, and eating behaviors [15].

Our study is the first to analyze patients’ reasons for undergoing bariatric surgery merged with quantitative data from the SOReg. Three quarters of the sample were women and one quarter men, which is representative of the patient population undergoing bariatric surgery in Sweden [16].

Other studies with the aim to investigate reasons for undergoing bariatric surgery report similar results to ours, showing that improved health or an improved medical condition is the main reason for many patients [6, 17–19]. Psychosocial health (appearance, self-image, social interaction) can also be a primary reason for some to undergo the surgery [6, 17]. In our study, appearance was stated as a reason by 27% of patients, with no differences between the sexes. The wish by patients to improve their medical condition was the most common reason reported in this study (59%), followed by physical limitations making day to day life difficult (42%).

Subgroup analyses highlighted significant differences between women in different age groups, with younger women being more distressed over their appearance than older women. Weight stigma has been highlighted as a threat to physical and mental health [20]. In the US, weight discrimination is common, especially in women and younger persons [21]. Women tend to suffer more from weight stigma, which impacts employment and education to a greater extent than for men with obesity [22]. Previous research has shown that men tend to have higher BMI, more comorbidities, and more severe complications after surgery, but still experience better mental well-being than women [23].

In this study, there were no significant sex-related differences in reasons for undergoing bariatric surgery. Lack of gender differences was also reported in a recent study likewise investigating patients’ motivation and goals for undergoing bariatric surgery [19]. By contrast, Libeton et al (2004) report that more women reported appearance, while men cited medical condition as motivation [14]. However, this may be because patients were told to reflect on their reasons for seeking surgery in the light of statements designed by the authors. Libeton et al’s and our results may perhaps be due to a hidden gender disparity and that men do not talk about their true reasons for undergoing surgery. However, our results indicate that Swedish men can in fact indicate that appearance or embarrassment is a reason for undergoing surgery. The trajectory of choosing to undergo bariatric surgery, and subsequent postoperative recovery, may differ between men and women. It is important that research on possible gender differences is ongoing and that clinicians are aware of these differences.

Health-related QoL after bariatric surgery has been carefully examined in previous research [24]. Our results confirm the path for HRQoL, where the PCS score increases after surgery [8, 24]. The most frequent reasons for undergoing surgery were medical conditions and physical limitations, which are included in the PCS [25, 26]. Interestingly, the MCS showed an increase 1 year after surgery but a decrease at 2 years after surgery. This is also confirmed by others [24, 27], although one study reported no improvement in MCS score after surgery [28]. Health-related QoL has previously been associated with weight loss [9, 29], but this association was not explored in our study.

In the study by Libeton et al [14], employment prospects were reported by patients as an important reason for wanting to undergo bariatric surgery. In the present study, 9% of patients gave employment prospects as a reason for wanting to undergo the surgery. Also, the employment-related statement showed the highest MCS values preoperatively and postoperatively. This contrasts with the results described by Antonsson et al, who found that persons on sick leave or job seekers reported lower results in all dimensions of the SF-36 [30]. However, the aim for our study was not to investigate employment and its relation to HRQoL; rather, it was to explore reasons for, and possible associations with, undergoing surgery. Although some participants in this current study were job seekers or on sick leave, only a few considered employability as a reason for undergoing surgery.

This study is not without limitations. The results may have been somewhat different if someone else had performed the deductive analysis. However, the initial codes were easy to sort into the chosen statements. We report quotes and expressions and terms the patients used to enhance transparency. We have chosen to explore reasons for surgery and possible associations. If patients had had predefined statements to choose from, or if patients had been instructed to rank their reasons our results may have been different. This area has not previously been thoroughly studied and research needs to be ongoing to learn more about patients’ reasons, expectations, and motivational factors for wanting to undergo bariatric surgery.

## Conclusion

The main reasons reported for undergoing bariatric surgery were a wish by patients to improve their medical condition and the will to make day to day life easier. There were few sex differences, but age affected patients’ reasons for wanting to undergo the surgery. Health-related QoL trajectories improved regardless of reasons, although the MCS score leveled out or decreased at the 2-year follow-up.

## Supporting information

S1 Table Mean weight in different categories, presented for the whole sample, and differences between men and women. No statistically significant differences between groups or stratified for sex.
(DOCX)

## Abbreviations

BMI: body mass index
EBMIL: excess body mass index loss
HRQoL: health-related quality of life
MCS: the mental component summary
PCS: physical component summary
SD: standard deviation
SG: Sleeve gastrectomy
SOReg: the Scandinavian Obesity Surgery Registry
