# Supplementary material for: Do reasons for undergoing bariatric surgery influence weight loss and health-related quality of life?–A Swedish mixed method study
Source: PLoS One. 2022 Oct 10;17(10):e0275868. doi: 10.1371/journal.pone.0275868 (PMC9550063; doi:10.1371/journal.pone.0275868)
Supplement: S1 Table — No statistically significant differences between groups or stratified for sex. (DOCX) [file pone.0275868.s001.docx]

S1 Table: Mean weight in different categories, presented for the whole sample, and differences between men and women. No statistically significant differences between groups or stratified for sex.

|  | Weight preoperatively, kg,  mean (SD) | Weight loss  1 year after surgery, kg,  mean (SD) Lost to follow up n=27 | Weight loss  2 years after surgery, kg,  mean (SD)  Lost to follow up n=41 |
| --- | --- | --- | --- |
| **Appearance:** I am distressed by my physical appearance, and I feel the need to improve it (n=182).  Men (n=33)  Women (n=149) | 118.64 (20.49)  139.91 (19.04)  113.93 (17.66) | -40.12 (11.85)  -45.69 (15.54)  -38.86 (10.51) | -39.38 (12.97)  -43.37 (16.87)  -38.52 (11.87) |
| **Medical condition:** I want to improve medical conditions associated with my obesity (n=408).  Men (n=86)  Women (n=322) | 118.61 (19.60)  136.86 (17.37)  113.73 (17.15) | -39.39 (11.65)  -41.41 (14.70)  -38.84 (10.65) | -38.54 (12.53)  -39.13 (14.40)  -38.38 (11.98) |
| **Physical fitness:** I lack physical fitness and want to be more active to enjoy life more (n=280).  Men (n=51)  Women (n=229) | 118.40 (19.36)  140.24 (21.02)  113.54 (15.20) | -39.49 (11.16)  -43.59 (14.29)  -38.60 (10.17) | -38.86 (12.41)  -42.36 (15.21)  -38.14 (11.66) |
| **Health concerns:** I am concerned that my health will deteriorate, and my life will be shortened (n=266).  Men (n=56)  Women (n=210) | 119.48 (21.25)  139.14 (24.64)  114.23 (16.78) | -39.32 (12.58)  -43.04 (16.86)  -38.38 (11.08) | -38.48 (13.56)  -39.67 (16.43)  -38.18 (12.75) |
| **Embarrassment:** I am embarrassed socially about my weight (n=86).  Men (n=14)  Women (n=72) | 120.71 (18.66)  139.29 (11.41)  117.10 (17.65) | -40.52 (11.74)  -47.15 (10.87)  -39.21 (11.53) | -40.16 (12.78)  -49.43 (10.01)  -38.20 (12.49) |
| **Physical limitation:** I feel that my physical limitation of obesity makes day to day living very difficult (n=288).  Men (n=60)  Women (n=228) | 119.98 (21.99)  140.18 (23.87)  114.66 (18.08) | -40.56 (12.72)  -45.62 (16.61)  -39.21 (11.10) | -39.34 (13.98)  -41.30 (17.49)  -38.86 (12.98) |
| **Employment prospects:** I want to improve my work ability and/or improve my employment prospects (n=65).  Men (n=21)  Women (n=44) | 123.72 (23.75)  141.48 (26.00)  115.25 (17.24) | -41.52 (11.93)  -45.95 (11.59)  -39.35 (11.62) | 40.33 (14.53)  -44.33 (13.51)  -38.33 (14.76) |

SD = standard deviation.
